# Towards environmentally sustainable human behaviour: targeting non-conscious and conscious processes for effective and acceptable policies

**DOI:** 10.1098/rsta.2016.0371

**Published:** 2017-05-01

**Authors:** Theresa M. Marteau

**Affiliations:** Behaviour and Health Research Unit, Institute of Public Health, University of Cambridge, Cambridge CB2 0SR, UK

**Keywords:** behaviour change, non-conscious processes, acceptability

## Abstract

Meeting climate change targets to limit global warming to 2°C requires rapid and large reductions in demand for products that most contribute to greenhouse gas (GHG) emissions. These include production of bulk materials (e.g. steel and cement), energy supply (e.g. fossil fuels) and animal source foods (particularly ruminants and their products). Effective strategies to meet these targets require transformative changes in supply as well as demand, involving changes in economic, political and legal systems at local, national and international levels, building on evidence from many disciplines. This paper outlines contributions from behavioural science in reducing demand. Grounded in dual-process models of human behaviour (involving non-conscious and conscious processes) this paper considers first why interventions aimed at changing population values towards the environment are usually insufficient or unnecessary for reducing demand although they may be important in increasing public acceptability of policies that could reduce demand. It then outlines two sets of evidence from behavioural science towards effective systems-based strategies, to identify interventions likely to be effective at: (i) reducing demand for products that contribute most to GHG emissions, mainly targeting non-conscious processes and (ii) increasing public acceptability for policy changes to enable these interventions, targeting conscious processes.

This article is part of the themed issue ‘Material demand reduction’.

## 1. Introduction

Climate change is being driven by excess greenhouse gas (GHG) emissions arising from a range of human activities including production of bulk materials (e.g. steel and cement), use of energy from non-sustainable supplies (e.g. fossil fuels) and production of animal source foods (particularly ruminants and their products). Effective strategies for timely reduction of the demand leading to these damaging levels of production and supply require systems thinking involving contributions from many disciplines. Evidence needs to be generated for multiple interventions implemented at different points in transformed supply and demand chains within the complex systems that comprise the multi-dimensional environments in which we live. The kinds of transformations being called for include action in seven dimensions (science, law, technology, money, democracy, culture and behaviour) [1], a circular economy in which prosperity is privileged over growth [2], and where the most sustainable product is the one never bought in the first place [3].

The focus of this paper is upon one contribution to these envisaged transformations, namely that of changing behaviour across populations to reduce consumption to a sustainable level. Drawing upon evidence for reducing over-consumption of products that harm health (principally tobacco, alcohol and sugar) this paper considers, first, the types of interventions likely to be effective at reducing demand for products that contribute most to carbon and equivalent emissions, and second, how public acceptability for these interventions might be increased to enhance their chances of being incorporated into policy.

## 2. Understanding and changing human behaviour

### (a) Values and sustainable behaviour

Much of the research published to date on sustainable consumption has focused on interventions targeting individual behaviour change [4] with a particular focus on values as predictors and targets for change (e.g. [5,6]). This has led to a wide range of interventions aimed at either changing values or activating existing values. Primarily, these involve conveying information about climate change and the potential harms and benefits of different behaviours. Behaviours that have been targeted include household energy use, recycling and food waste. While some change has been reported, the scale of change is insufficient to make a discernable impact on behaviour at the level needed [7,8]. This is in keeping with the broader psychological literature on behaviour and behaviour change. While our values are reflected in some of our behaviours some of the time, the strength of this association is variable and often weak. It is strongest for infrequently performed behaviours such as voting in a national election or selecting an energy supplier and weakest for routine or habitual behaviours which comprise many of the targets for unsustainable consumption behaviours [9]. Even when values are stronger predictors of behaviour targeting values to change the behaviour is not always the most effective way of achieving change, as discussed below.

### (b) Models of behaviour

*‘Essentially, all models are wrong but some are useful.’* [10, p. 424].

Many models have been described for understanding human behaviour from a range of disciplines including psychology, economics, sociology and social anthropology. The focus of this section is upon psychological models and in particular those that highlight the role of physical environments in shaping behaviour, often without our awareness. Economic environments also play a key role in shaping behaviour, described briefly at the end of this section.

For much of the twentieth century the dominant models of behaviour and behaviour change in both economics and psychology had their roots in subjective expected utility models (SEU) [11]. These models predict choices based on the option with the highest expected utility, derived from judgements about the personal utility of the outcome and the likelihood of the outcome occurring. By the end of the twentieth century the limited effectiveness of interventions based on models that characterized behaviour as representing reflective, conscious choices was becoming apparent [12,13]. For example, the results of a meta-analysis of the experimental evidence for a causal link between intentions and a wide range of behaviours led the authors to conclude: ‘… intentional control of behaviour is a great deal more limited than previous meta-analyses of correlational studies have indicated’ [9].

Dual-process models of behaviour go some way towards addressing these limitations of SEU-based models. Dual-process models describe two sets of processes, operating in parallel, with one set being largely reflective, conscious and reason-driven, and the other set being largely automatic, non-conscious and emotion-driven. The details of the two systems, including their names, vary between models. They include ‘System 1’ versus ‘System 2’ [14], ‘Hot’ versus ‘Cold’ [15] and ‘Reflective’ versus ‘Impulsive’ [16], but the core characteristics of the two systems are essentially the same. The systems operate in parallel and are most often synergistic, i.e. working in concert to allow goals to be met, with the faster system supporting performance of routine or habitual behaviours, such as travelling to work, thereby freeing up our limited cognitive capacity to allow the slower more deliberative system to support behaviours requiring reflection such as reading a newspaper. The systems do, however, sometimes work antagonistically, such as when an individual with a strong intention to lose weight nonetheless finds they bought the tempting bar of chocolate that was displayed at the till where they bought their newspaper. Importantly, most of our behaviour is under the control of the faster, automatic system so effective strategies for reducing consumption will involve mainly targeting this system.

**Figure 1.**
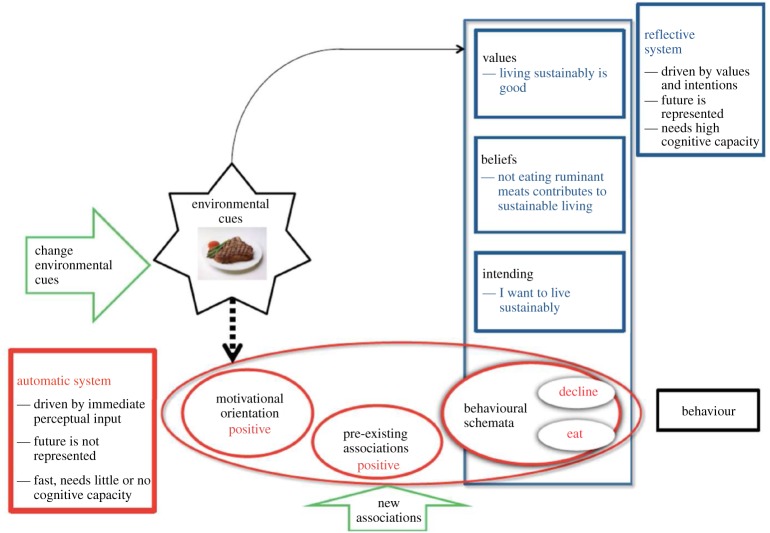
Automatic and reflective systems guiding behaviour, with potential points for intervention (after Strack & Deutsch [16])*.* (Online version in colour.)

The reflective impulsive model [16] is one of the most specified and is used to illustrate some of the key characteristics of these models and their implications for changing behaviour. A simplified version of the model is presented in figure 1 to outline possible responses to the offer of a beef-steak depending on the relative activation of the reflective or the automatic systems.

Imagine sitting at a conference dinner. You are not talking to anyone and are wondering what food is to be served. You are offered a beef-steak. You start to think about the environment as your reflective system is activated. You rehearse the importance for you of trying to live sustainably that includes not eating beef. You therefore decline the steak and instead dine on the chips and salad. You then join in the lively conversation that has started up around you about a controversial talk given earlier in the day.

Imagine now that you are at the same conference dinner and your attention is taken up with an interesting conversation with your neighbour about a talk you both attended. You are offered the beef-steak. The perception of the steak rapidly and imperceptibly activates your automatic system. Your immediate, automatic response to the steak is positive resulting in the activation of a behavioural response which is to eat the steak.

In addition to illustrating how the same cue can result in different responses in the same individual depending on which behavioural control system is activated, figure 1 also illustrates two points for intervening to make a particular behavioural option—in this case not eating the beef-steak—more likely. These include altering the environmental cue by serving a meal with lower GHG emissions. This would be mainly plant-based but might include some pork or chicken—the ‘climate carnivore diet’ with lower GHG emissions than a vegetarian diet [17]. The second intervention point involves altering pre-existing positive associations with the beef-steak perhaps through labelling the food on the menu to highlight its negative associations with population health (including increased risk of bowel cancer) or planetary health (including rise in global warming beyond 2°C).

Further examples of interventions are described below using the two points for intervening in the automatic system, as illustrated in figure 1. This is merely illustrative and is not based on a systematic search or summary of existing evidence.

*Changing environmental cues* to activate behavioural schemata for sustainable behaviours with pre-existing positive associations.

One example of this concerns the presentation to consumers of energy supplies. For many years, despite saying they would use green energy if offered a choice, fewer than 1% of Germans made this choice [18]. But when a green energy supply was offered as a default, 69% ended up with this energy supply, compared with 7% not defaulted to this supply [19]. In addition to altering defaults, making more sustainable options visually prominent can shape behaviour. For example, in a behavioural experiment designed to increase selection of meat-free meals in a college cafeteria, altering the default shown on short menus to include just five meat-free meals resulted in 90% of selected meals being meat-free compared with just 40% in the control condition [20]. Providing information about the environmentally friendly nature of meat-free meals had no impact.

*Creating new automatic associations* with products or behaviours so that sustainable ones have stronger positive associations.

Creating new automatic associations generally requires multiple pairings or presentations of a product or a behaviour with a novel stimulus. There is, however, some evidence that using images or symbols to reduce the appeal of a product or behaviour can occur after just a few pairings [21–23]. By contrast, increasing appeal with just a few pairings seems less effective [23,24]. In keeping with this, preliminary evidence suggests that presenting the environmental harms of purchases is more effective in shifting preferences away from unsustainable products than presenting the environmental benefits of sustainable products, effects unrelated to level of environmental concern [25]. By contrast, labels that denote the sustainability of a product do not appear to influence consumption [26].

The difficulty of altering pre-existing associations with unsustainable behaviours is not to be underestimated given the ingrained positive cultural associations with many behaviours and products that contribute to GHG emissions. These include buildings and infrastructure projects displaying gleaming steel and cement structures, warm homes and shopping centres, high performance cars, and delicious cheeses and meats. That said, the associations with tobacco smoking have changed dramatically over the last 75 years. Once a product associated with glamour and sex, promoted by doctors as health enhancing, in a growing number of countries it is now more strongly associated with poverty and premature, painful death. Achieving such a shift involved multiple interventions over time in the face of fierce opposition from the tobacco industry, an opposition that continues today [27]. Interventions that contributed to this shift in the UK included bans on the marketing of cigarettes, starting with advertising on TV, cinema, newspapers and magazines to the most recent ban on the selling of tobacco in branded packs.

### (c) Changing economic environments to change behaviour

In addition to changing cues in physical environments, changing economic environments principally through price changes is another component of effective strategies to reduce consumption. This is most clearly seen in health contexts for tobacco but also alcohol and increasingly for reducing consumption of sugar [28–30]. Increasing prices of tobacco and sugar has been particularly effective in reducing their consumption among the poorest, a group that consume more of these two products [31,32]. While price is used to encourage reduced consumption of products that harm the environment such as reduced taxes on the use of fuel-efficient cars and a levy on the use of plastic bags, with proposals for a tax on meat [33], proposals from macro-economists for an international price on carbon at the scale needed to address the scale of global warming have yet to be implemented (e.g. [34,35]). As discussed in §3 on increasing public acceptability of interventions to change behaviour, increasing the price of products is the least popular intervention for policy-makers, the public and industry. An exception to this is the levy on plastic bags introduced in several countries, considered the most popular tax in Europe [36]. While this has proved a dramatically effective tax in changing behaviour, reducing use by 80% or more, reducing plastic bag use has a very small impact on GHG emissions. In addition, current evidence suggests that this change does not ‘crowd in’ other sustainable behaviour [37]. The importance of researchers and policy-makers targeting behaviours that contribute most to reduced GHG emissions is discussed below.

### (d) Strengthening research on changing sustainable behaviours

Recent overviews of interventions to achieve sustainable consumption reveal an increase in research activity albeit from a low baseline (e.g. [18,38]). These reviews also reveal a shift in interventions being developed and evaluated, away from those targeting conscious processes and towards those based on stronger behavioural science, with potential to change behaviour at the scale needed [7,39]. This burgeoning research would be strengthened further by drawing upon lessons learned from the larger, more developed research on changing health-related behaviour across populations. These concern the behavioural focus of interventions, study designs and quality.

#### (i) Behaviours to target for interventions

Targets for change need to be those that have the potential to make the largest impact on reducing carbon and equivalent emissions. For example, there has been more research and policy interest in reducing food waste than on reducing meat consumption, the former contributing an estimated 1–2% to emissions and the latter an estimated 50% [17]. Similarly, there has been more research and policy focus on reducing use of plastic bags than on reducing use of other materials [40]. The development of stronger links between environmental and behavioural scientists could shift the focus of behavioural science intervention development and evaluation to encompass the behaviours that contribute most to carbon and other emissions.

#### (ii) Types of studies

(a) *Reviews*. The strength of evidence for interventions to achieve sustainable behaviours would be increased by more systematic literature reviews including formal evidence synthesis where possible to estimate the effect sizes of interventions. While reviews exist, these have mainly used unsystematic methods, with results synthesized narratively. (An example of the type of review needed is one on the use of feedback to reduce energy consumption [41].) Without systematic reviews it is difficult to know, first what is already known about the effectiveness of a particular intervention, and second, where research gaps lie. Review methods developed within the Cochrane collaboration can readily be applied to this field (http://training.cochrane.org/handbook).

(b) *Experiments*. The second type of study that is key to increasing the strength of evidence in this area is the field experiment, i.e. a comparison between two or more groups that differ only in the intervention with allocation to groups determined if at all possible by randomization. While such designs are increasingly evident in research on interventions for sustainable behaviour, good quality experiments are scarce.

#### (iii) Quality of studies

Three ways in which experimental studies would be improved concern outcome measures, study populations and study power. Outcomes need to be objectively measured behaviours and not self-reported behaviour change or intentions to change behaviour, both of which are subject to bias including over-estimation of change. Study populations need to be representative of general populations. While proof-of-concept or pilot studies can usefully rely on convenience samples such as students, responses from these and other highly educated populations can differ markedly from general population responses to the same intervention (e.g. [42]). In addition, experiments need to be powered to detect expected effect sizes to guard against potentially impactful interventions being dismissed.

### (e) Implementing research findings

Having identified key drivers and effective interventions, effective implementation of many of them will be through policies that require regulation [35,43]. This is a key challenge, described in one report in the following way: ‘Lack of progress on climate change is caused by this mixture of vested interests, political paralysis and civic ambiguity’ [44].

The next section considers how public acceptability of interventions to change behaviour might be increased to overcome these barriers to progress.

## 3. Increasing public acceptability for changing behaviour: targeting conscious processes

Implementing many of the interventions described in the previous sections will require regulation. This will probably include increasing prices and restricting marketing of products and activities with high carbon and equivalent emissions such as animal source foods, cement, aluminium and plastic materials, car and plane travel. Mandated labelling schemes might also be introduced. Other interventions include capping the proportion of non-renewal energy that companies can supply.

Such changes will meet with strong opposition from industries that stand to lose. In health contexts, the tobacco, alcohol and food industries have lobbied and framed debates on regulation often with great success to the detriment of population health [19,45–48]. Similar tactics have been described for fossil fuel related industries [49].

Public acceptability is a critical consideration for policy-makers. Public acceptability also goes some way towards addressing ethical concerns about targeting non-conscious processes to change behaviour [50]. Paradoxically, public acceptability is high for information-based interventions which are generally of very limited effectiveness in changing behaviour of the type and scale needed for sustainable consumption, and lowest for price-based and other interventions generally of higher effectiveness [51,52]. This pattern is evident in a survey of six European nations assessing acceptability of a range of interventions targeting population health as well as the environment [18]. For example, education campaigns targeting childhood obesity were supported by 87% of respondents, with fewer (64%) supporting removal of sweets at supermarket checkouts. On environmental policies, 68% supported energy companies encouraging defaulting customers into green energy providers (with 4% fewer than this supporting mandating this, a far more effective intervention), but just 36% supported an airline carbon tax, a potentially far more effective intervention both in terms of changing behaviour and impact on the environment.

One possible resolution to this paradox of public acceptability generally being lower for more effective interventions and higher for ones that are less effective stems from the observation that public perception of intervention effectiveness is one of the most reliable predictors of acceptability [53–56], with some evidence suggesting a causal link [57,58]. For example, public acceptability for each of five interventions for tackling obesity was most strongly predicted by perceived effectiveness in both US and UK samples [56]. Public acceptability of interventions for smoking cessation, weight loss [59] and reduced alcohol consumption [3] increased with quantification of their effectiveness.

In the context of environmentally sustainable behaviours, willingness to change one of five sets of behaviours, including driving less and eating less meat, increased with perceived effectiveness of the action [60]. A series of studies comparing support for health (smoking) and environmentally focused interventions (water and energy) based on automatic, non-conscious (‘System 1’) or reflective, conscious (‘System 2’) systems revealed more support for the latter. Support for System 1 interventions (although not System 2 ones) increased with an assertion of its greater effectiveness, with and without evidence quantifying the effect [18].

Public acceptability of interventions might therefore be increased by effective communication of the impact of interventions targeting physical and economic environments to reduce carbon emissions. Building on existing research on personal risk communication [61,62], the precise content and formats for effectively communicating evidence about population and planetary level outcomes of carbon emissions await investigation.

There are two further factors that may be important to target in increasing policy acceptability. The first factor is people's understanding of the non-conscious nature of much of human behaviour. For example, the more people attributed over-consumption to the physical environment in which the behaviour occurs, but not free will, the greater their support for policy interventions to tackle obesity [56]. The second factor is the value attached to the outcomes of intervention. Reflecting a higher value attached to benefits than risks, sustainable policies attracted greater support when outcomes were presented in terms of the health benefits of mitigation rather than the health risks of climate change [63]. Outcomes of reducing GHG emissions that are valued by significant proportions of the population are more difficult to find than those in relation to such health threats as obesity particularly in children [51]. For example, just 5% of the UK population cite the environment as an important issue facing Britain today (Ipsos-MORI Issues Index August 2016). This reflects the nature of the threat which is perceived as abstract, uncertain, far in the future as well as one on which they can have little impact [44].

**Figure 2.**
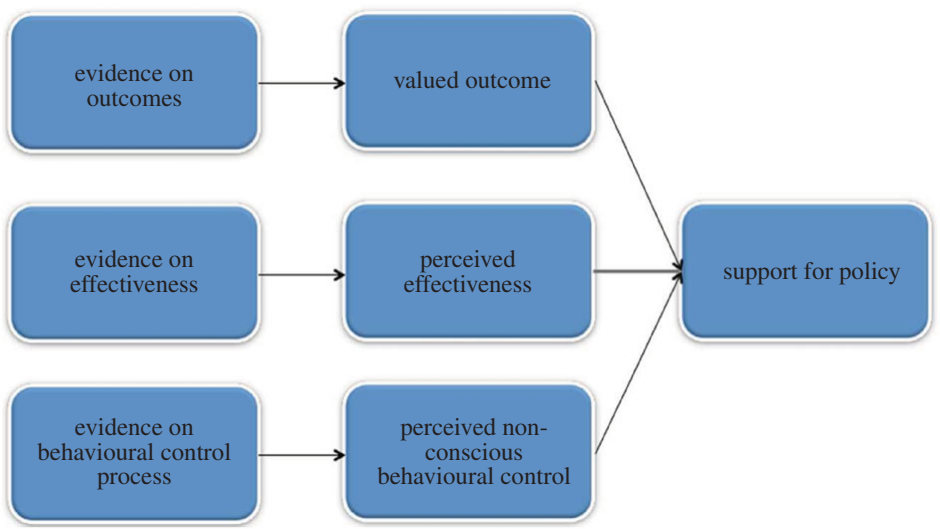
Possible pathways to increasing support for policies targeting behaviour change. (Online version in colour.)

Figure 2 shows the three factors described above that are associated with support for policies focused on changing behaviour across populations. The extent to which these variables are amenable to change, through effective, salient communications, awaits testing. Also awaiting testing is the extent to which any change in these variables has a corresponding change on policy support.

In addition to communications aimed at increasing public acceptability, a range of other interventions has been described for increasing public demand for policy action. These include interventions aimed at establishing social movements involving non-government organizations with the resources to be able to act including countering industry interests that conflict with policy goals [64]. Two case studies illustrate attempts to implement policies to reduce consumption of sugary drinks, with different outcomes. The first concerned an attempt to introduce a 16 ounce (454 ml) limit on the size at which sugar sweetened beverages could be sold in food outlets in New York City. This met with much resistance and was ultimately unsuccessful [65]. A newspaper survey of New York residents reported 60% opposed the proposed 16 ounce cap. The survey was conducted during a media campaign, funded by soda manufacturers highlighting the rights of citizens to purchase soda in sizes ‘without interference from bureaucrats’. This may have influenced responses. Subsequent surveys show more support for this policy [56].

Legislation to implement a sugary drinks tax in Mexico is an example of a successful change of policy with preliminary evaluation showing the expected outcomes on reduced consumption of sugary drinks [32]. With funding from Bloomberg Philanthropies, non-governmental organizations engaged in a range of activities to garner public support for the tax including buying prominent advertising space to counter industry opposition to sugary drinks taxes.

## 4. Concluding comment

Huge and immediate changes are needed to reduce demand for environmentally unsustainable products in order to meet a climate change target of 2°C. Towards this a step change is needed in the number and quality of behavioural experiments evaluating the impact of altering cues in physical environments to achieve sustainable behaviours. Evidence on effective interventions is generally insufficient for their implementation. Developing research on increasing public acceptability of effective interventions including economic ones could raise the chances that policy-makers act in line with the growing evidence for achieving sustainable behaviours to meet the unprecedented global challenge from climate change.
